# Comparison of Environmental DNA Metabarcoding and Underwater Visual Census for Assessing Macrobenthic Diversity

**DOI:** 10.3390/biology14070821

**Published:** 2025-07-06

**Authors:** Zifeng Zhan, Weiwei Huo, Shangwei Xie, Wandong Chen, Xinming Liu, Kuidong Xu, Yanli Lei

**Affiliations:** 1 Laboratory of Marine Organism Taxonomy and Phylogeny, Qingdao Key Laboratory of Marine Biodiversity and Conservation, Institute of Oceanology, Chinese Academy of Sciences, Qingdao 266071, China; zzhan@qdio.ac.cn (Z.Z.); h2580852@outlook.com (W.H.); kxu@qdio.ac.cn (K.X.); 2 School of Marine Science and Engineering, Qingdao Agricultural University, Qingdao 266109, China; 3 University of Chinese Academy of Sciences, Beijing 100049, China; 4 Nanji Islands National Marine Nature Reserve Administration, Wenzhou 325400, China; shangweixie2025@163.com (S.X.); chen_0636@sina.com (W.C.); 5 Institutes of Marine Drugs, Guangxi University of Chinese Medicine, Nanning 530200, China; liuxm@gxtcmu.edu.cn

**Keywords:** eDNA, species detection efficiency, marine protected area, subtidal zone, biodiversity assessment

## Abstract

The rapid advancement of environmental DNA (eDNA) technology has transformed ecological research, particularly in aquatic ecosystems. However, the optimal sampling matrix (e.g., water or sediment) and the potential for eDNA to replace or complement traditional underwater visual census (UVC) remain unclear. Here, we integrate water eDNA, sediment eDNA, and UVC approaches to systematically compare the diversity of benthic macrofauna in the subtidal zones of the Nanji Islands, China. Our results show that sediment eDNA samples exhibited the highest species richness, while UVC had the lowest. Each method revealed distinct species profiles, with relatively few shared taxa at the order level and below. Environmental eDNA showed significant advantages in detecting key phyla such as Annelida and Arthropoda. In contrast, traditional UVC was crucial for identifying certain taxa, such as Bryozoa, which were undetectable by eDNA methods. The low overlap in species detected by these methods underscores their complementary nature, highlighting the necessity of integrating multiple approaches to achieve a more comprehensive and accurate biodiversity assessment. Future research should focus on refining eDNA techniques, such as developing more universal primers, to further enhance their applicability in biodiversity monitoring.

## Simple Summary

This study evaluates the efficacy of environmental DNA (eDNA) metabarcoding and underwater visual census (UVC) in assessing the diversity of subtidal macrobenthic communities. We compared water eDNA, sediment eDNA, and traditional UVC methods in the Nanji Islands, China. Sediment eDNA demonstrated superior performance in detecting key benthic phyla such as Annelida and Arthropoda, whereas UVC was more effective for large and active organisms. Integrating these methods provides a more comprehensive biodiversity assessment, highlighting the importance of combining molecular and traditional techniques for effective conservation and management strategies in marine ecosystems.

## 1. Introduction

The subtidal zone, a critical interface between intertidal zones and shallow sea, usually harbors a rich diversity of benthic fauna, yet it is one of the areas most severely affected by human activities [1]. Traditional in situ observations through underwater visual census (UVC)—where trained SCUBA divers survey species richness and community assemblages in the field—have long been the predominant method for studying marine organisms [2]. However, this approach is limited by the need for specialized skills, suitable field conditions, and significant resources [3]. Moreover, the complex currents and topography of subtidal zones often pose substantial challenges for UVC surveys, restricting the comprehensive biomonitoring of these habitats [4].

Recent advancements in environmental DNA (eDNA) technology have revolutionized biodiversity assessments in aquatic ecosystems [5]. eDNA metabarcoding allows for the non-invasive detection of species through the analysis of genetic material present in environmental samples such as water and sediment [6]. This approach has been successfully applied to monitor fish and invertebrate communities in various aquatic habitats, including rivers, lakes, and marine environments [5,6,7,8]. However, the optimal sampling matrix (water vs. sediment) and the potential for eDNA to replace or complement the traditional UVC method to access the macrobenthos diversity remain uncertain.

The Nanji Islands, located in the eastern sea area of Zhejiang Province, China, is a biosphere reserve of the United Nations Educational, Scientific and Cultural Organization (UNESCO). This subtropical marine protected area is characterized by complex subtidal zones with high biodiversity [9]. The seabed terrain in this area slopes downwards from northwest to southeast, with a water depth generally between 15 and 25 m. There are two deep-water channels on the northeast and southwest sides of Nanji Island, with a depth of over 30 m and a maximum depth of 45 m. The subtidal areas here include several distinct habitat types, such as steep rocky cliffs, platforms, boulder fields, small area beaches, and sediments mainly composed of silty clay. Due to the fact that the subtidal zone with a depth less than 20 m is mainly composed of rocks, bottom trawl survey is impossible, and box-type mud collection is also limited. While the underwater visual census (UVC) is the remaining available traditional method, a systematic biodiversity survey is still lacking.

Generally, the Nanji Islands provide an ideal setting for comparing the effectiveness of different biodiversity assessment methods. In this study, we integrate water eDNA, sediment eDNA, and UVC methods to systematically compare their efficiency in detecting benthic macrofaunal diversity in the subtidal zone with a depth < 20 m of the Nanji Islands. Our objectives are to quantify the differences in species detection efficiency among the three methods, and explore the complementary nature of these methods across different taxonomic levels.

## 2. Materials and Methods

### 2.1. Study Area and Sample Location

**Figure 1:**
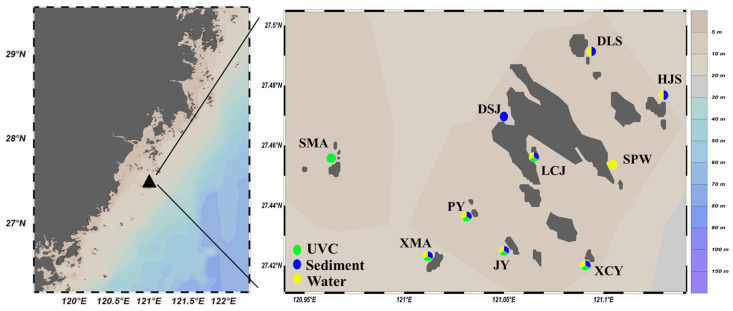
Sampling sites in the subtidal zone of the Nanji Islands (The capitalized names in the right-side figure are abbreviations for each sub-island. “SMA” stands for “Shangmaan”, “XMA” for “Xiamaan”, “PY” for “Poyu”, “JY” for “Jianyu”, “XCY” for “Xiaochaiyu”, “LCJ” for “Longchuanjiao”, “DSJ” for “Dashanjiao”, “SPW” for “Sanpanwei”, “HJS” for “Houjishan”, and “DLS” for “Daleishan”).

The Nanji Islands (27°27′43″ N–27°46′48″ N, 120°02′55″ E–121°07′58″ E) are located in the eastern sea area of Wenzhou City, Zhejiang Province, China. This subtropical marine protected area is characterized by mainly rocky inter- and sub-tidal zones with high biodiversity. Ten subtidal sites representing the broader subtidal environment were selected for this study based on accessibility by boat on 8–10 May 2024 (Figure 1).

### 2.2. Underwater Visual Census (UVC) and Photo Analysis

UVC survey conducted during high-tide period. The basic unit of UVC was a 50 m long transect line, with macrobenthos surveyed in two 1 m wide by 2 m high bands on either side of the transect line [10]. The proportions of the area examined in relation to the rocky subtidal area of each site ranged from approximately 10% to 30%. Digital photo-quadrats were taken at 2.5 m intervals along the transect line (i.e., 20 per 50 m transect). Due to the extremely low transparency of seawater (<1 m), the SMA, DSJ, HJS, and DLS sites were not suitable for diving. Consequently, the UVC units of only six stations with a depth range of 5–15 m were completely investigated (Figure 1, marked by green color). Two taxonomists (W. Huo and W. Chen) estimated the number of species from the visual data together, without prior knowledge of the eDNA outcomes. Due to the generally low transparency of seawater, many photo-quadrats were not very clear, and only 30 species could be identified to species level (Table A1).

### 2.3. Environmental DNA Sampling

Water and sediment samples were collected from ten stations in the subtidal zones of the Nanji Islands. Considering that the larvae of benthic organisms may float in surface water layers, 1 L of seawater was collected from both surface and bottom layers by divers to gather as much macrobenthos eDNA as possible. The samples were mixed thoroughly, and 1.5 L seawater was filtered [11] through a 0.22 µm mixed cellulose ester (MCE) membrane, and the filter paper was immediately transferred to a 1.5 mL cryopreservation tube and stored at −20 °C.

Sediment samples were collected using a grab sampler near the water sampling sites. At the sites XMA and XCY, however, the sediment could not be collected by the grab sampler due to the thin sediment layer. Instead, divers collected sediment using 50 mL centrifuge tubes in those sites. Approximately 20 g of surface sediment was placed into a sterile, enzyme-free sampling bag and stored at −20 °C until further analysis.

### 2.4. DNA Extraction and Sequencing

Environmental DNA was extracted from water sample filters using the DNeasy Power Water Kit (Qiagen, Hilden, Germany) and from sediment samples using the QIAamp PowerFecal Pro DNA Kit (Qiagen, Hilden, Germany), following standardized protocols to ensure high extraction efficiency and purity. The COI gene fragments were targeted and amplified using universal primers mlCOIintF (5′-GGWACWGGWTGAACWGTWTAYCCYCC-3′) [12] and jgHCO2198 (5′-TAIACYTCIGGRTGICCRAARAAYCA-3′) [13]. The PCR conditions were as follows: initial denaturation at 94 °C for 3 min, followed by 35 cycles of 94 °C for 30 s, 55 °C for 30 s, and 72 °C for 2 min, with a final extension at 72 °C for 5 min. Negative controls (DNA-free samples) were processed in parallel to ensure the reliability of the PCR. Sequence libraries were prepared using the TruSeq^®^ DNA PCR-Free Kit (Illumina, San Diego, CA, USA) and quantified by Qubit and Q-PCR. The libraries were sequenced as 250 bp paired-end reads on a NovaSeq 6000 platform.

### 2.5. Bioinformatics Analysis

Raw sequencing data were processed using DADA2 (version 2.15.2) to remove primer sequences and low-quality reads [14]. Amplicon sequence variants (ASVs) were clustered at 97% similarity using VSEARCH, and ASVs with less than 0.01% abundance across all samples were filtered out [15,16]. Taxonomic annotations were performed against the NCBI database, focusing on eukaryotic groups. Non-marine groups (e.g., Arachnida, Insecta, and terrestrial Craniata) were manually excluded from the ASV table [17].

### 2.6. Statistical Analyses

To comprehensively assess the species diversity based on the three methods, species richness, Shannon–Wiener diversity, and Pielou’s evenness indices were employed. Species richness and the relative ratio of species number at class, order, and family level were compared among UVC, sediment eDNA, and water eDNA methods. Venn diagrams were generated to assess overlapping taxa detected by the different methods. As the species diversity data from sediment and water eDNA did not meet the normal distribution assumption (Shapiro–Wilk test, *p* < 0.05) or the homogeneity of variance assumption (Bartlett test, *p* < 0.05), a nonparametric Kruskal–Wallis test was used to analyze intergroup differences in species diversity. Post hoc Dunn tests with Holm–Bonferroni correction for *p*-values were further conducted to evaluate species diversity differences across different methods.

In the analysis of the community structure, Jaccard distances were calculated based on species composition matrices, and non-metric multidimensional scaling (NMDS) was used to visualize community differences among the three methods. The reliability of the ordination was evaluated by the stress value (the closer the value is to zero, the higher the degree of fit). The clustering of samples was observed in the NMDS plot, and the significance of community structure differences among groups was verified by ANOSIM tests (based on Jaccard distances). All statistical analyses and figures were performed using R version 4.0.3, with plots created using the ggplot2 package (version 3.3.3) [18].

## 3. Results

### 3.1. Sequencing and UVC Results

Only eight of ten water and sediment eDNA samples were successfully amplified even after repeating the PCR experiments several times, respectively (Figure 1, successful amplification stations marked by yellow and blue colors, respectively). A total of 657,161 and 752,136 raw reads were obtained from water and sediment eDNA samples, respectively. After bioinformatics processing, 1879 and 7487 amplicon sequence variants (ASVs) were identified for water and sediment eDNA, respectively. The majority of ASVs (79.30% for water eDNA and 64.90% for sediment eDNA) were either taxonomically unassigned or belonged to non-marine groups. The remaining ASVs represented a diverse range of marine taxa, including 11 phyla, 22 classes, 47 orders, 72 families, 72 genera, and 94 species for water eDNA; and 14 phyla, 28 classes, 64 orders, 114 families, 125 genera, and 166 species for sediment eDNA. In contrast, UVC identified a total of nine phyla, 16 classes, 18 orders, 29 families, 29 genera, and 39 species based on photo-quadrat analysis. Overall, the three methods collectively identified 15 phyla, 38 classes, 89 orders, 179 families, 206 genera, and 284 species (Table 1 and Table A1).

### 3.2. Comparison of the Taxonomic Compositions Among the Three Methods

**Figure 2:**
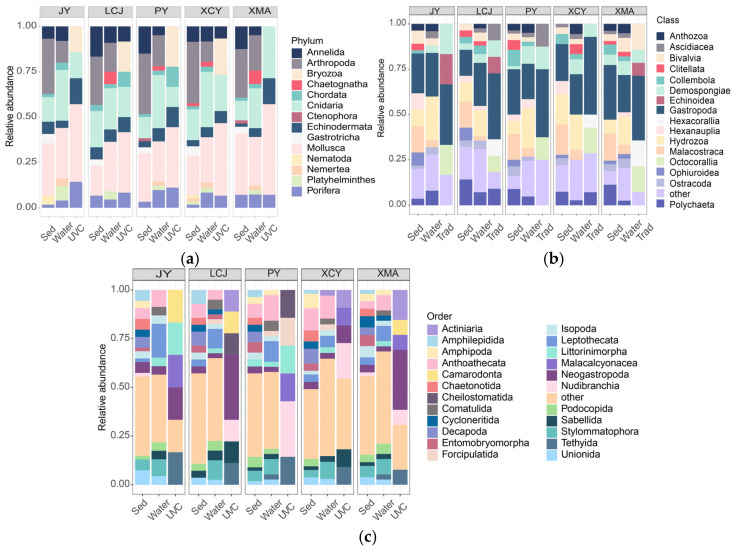
Stacked bar charts showing the percentage of species richness at the phylum (**a**), class (**b**), and order (**c**) levels for underwater visual census (UVC) and sediment (Sed) and water eDNA methods.

Taxonomic compositions were compared among the three methods at five common stations (Figure 2). Sediment eDNA detected the highest number of phyla (13), classes (27), and species (131), followed by water eDNA (10 phyla, 21 classes, 72 species) and UVC (seven phyla, 14 classes, 15 orders, 32 species). At the phylum level, sediment and water eDNA showed similar compositions, with Mollusca, Arthropoda, and Cnidaria being dominant (Table 1). In contrast, UVC identified Mollusca, Cnidaria, and Bryozoa as the most dominant group, and the Bryozoa was undetectable by eDNA methods (Figure 2a). At the class level, sediment eDNA was dominated by Gastropoda, followed by Malacostraca and Hydrozoa, while water eDNA showed a similar pattern with Gastropoda, Hydrozoa, and Demospongiae as dominant classes. UVC identified Gastropoda, Anthozoa, and Demospongiae as the dominant classes (Figure 2b). At the order level, sediment eDNA showed Anthomedusae, Decapoda, and Stylommatophora as dominant orders, while water eDNA was dominated by Leptothecata, Anthoathecata, and Stylommatophora. UVC identified Neogastropoda, Nudibranchia, and Demospongiae as dominant orders (Figure 2c).

**Figure 3:**
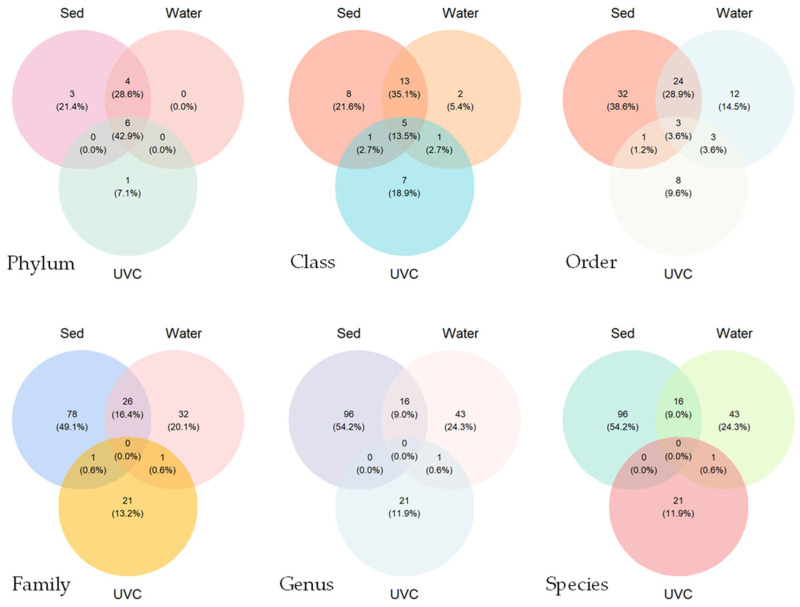
Venn diagrams showing the species overlaps at the different taxonomic ranks detected by underwater visual census (UVC) and sediment (Sed) and water eDNA methods at the common stations.

Venn diagrams revealed relatively high taxon overlaps at the phylum and class levels, and low overlaps at the order level and below among the taxonomic compositions from the three methods (Figure 3). Sediment eDNA detected the highest number of species overall, while traditional UVC methods detected fewer but unique species. No common species was found among the three methods. Only 18 common species were found between sediment and water eDNA samples, and only 1 common species was found between traditional UVC and sediment/water eDNA samples.

### 3.3. Alpha and Beta Diversity Among the Three Methods

**Figure 4:**
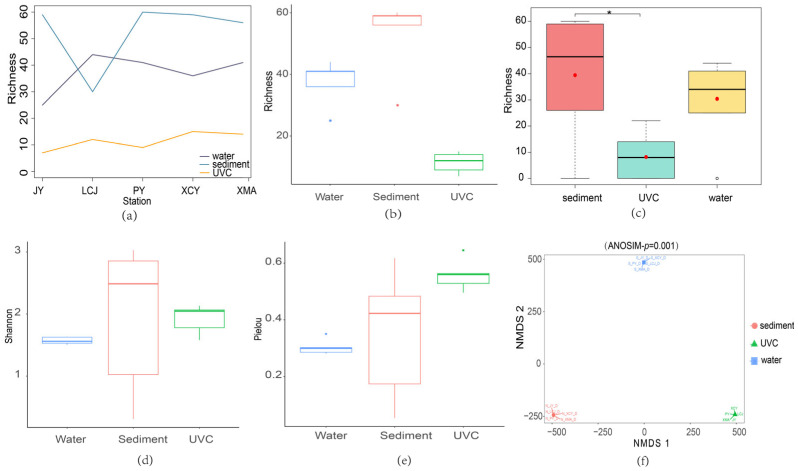
Alpha and beta diversity analyses based on sediment eDNA, water eDNA, and underwater visual census (UVC) methods. (**a**) Species richness based on the sediment eDNA, water eDNA, and UVC at the common sampling stations (JY, LCJ, PY, XCY, XMA). (**b**) The total species richness based on the three methods from all the common stations. (**c**) Significant richness differences among the three methods conducted by post hoc Dunn tests. *, indicating significant differences. (**d**) The total Shannon index based on the three methods from all the common stations. (**e**) The total Pielou’s evenness index based on the three methods from all the common stations. (**f**) The clustering of samples with non-metric multidimensional scaling (NMDS) plot.

Species richness was highest in sediment eDNA samples across all stations except LCJ, with UVC showing the lowest species richness (Figure 4a). Overall, sediment eDNA samples exhibited the highest average species richness, while UVC had the lowest (Figure 4b). The Kruskal–Wallis test and post hoc Dunn test indicated significant differences in species richness between sediment eDNA and UVC methods (Figure 4c). Sediment eDNA also consistently showed higher Shannon and Pielou’s evenness indices compared to water eDNA, indicating superior performance in both species abundance and community evenness (Figure 4d,e).

Non-metric multidimensional scaling (NMDS) ordination plots showed distinct clustering of samples from sediment eDNA, water eDNA, and UVC methods, with no overlap among the groups (Figure 4f). The stress value of zero and an ANOSIM test *p*-value of 0.001 confirmed the high reliability of the ordination results, indicating significant differences in community structure captured by the three methods. This suggests that each method provides unique insights into the biodiversity of the subtidal zone, highlighting the importance of integrating multiple approaches for comprehensive biodiversity assessment.

## 4. Discussion

### 4.1. Environmental DNA Is Essential for Biodiversity Assessment of Rocky Subtidal Zone

Traditional bottom trawling and grab sampling are unsuitable for biodiversity assessments in rocky subtidal zones. Underwater visual census (UVC) is often used for macrobenthic surveys, but its effectiveness is limited by low seawater transparency, as seen in this study. Compared to UVC, the present study demonstrates that eDNA is more effective for assessing benthic macrofaunal diversity, particularly for key phyla such as Annelida and Arthropoda (Table 1; Figure 3). The sediment eDNA detected a significantly higher species richness compared to the UVC method (Figure 4). Generally, both the sediment and water eDNA methods can uncover those relatively smaller benthos (e.g., Chaetognatha, Gastrotricha, Nemertea, Platyhelminthes, and most of Annelida and Arthropoda) that were missed by the UVC survey (Table 1 and Table A1). Furthermore, eDNA showed significant differences in community structure with the UVC (Figure 4f).

In terms of total number, the sediment eDNA detected more species than the water eDNA (166 vs. 94; Table 1). For all the phyla except Cnidaria and Chaetognatha, the sediment eDNA detected no less species than the water eDNA (Table 1). Notably, sediment eDNA failed to detect Octocorallia species, which were uncovered by water eDNA. Most octocorals prefer to live on hard substrates such as rocks, rather than sediments. Consequently, the octocoral eDNA in the sediment is too rare to amplify. In contrast, the eDNA concentration in water samples increases after filtration and concentration, which may contain more octocoral eDNA. For the planktonic Chaetognatha, it is not surprising that the water eDNA detected more species than the sediment eDNA (Table A1).

For the macrobenthos, the superior detection efficiency of sediment eDNA is likely due to the higher concentration and longer persistence of DNA in sediments compared to water samples, which allows for retrospective genetic monitoring and extends the seasonal window for species assessment [19,20,21,22,23,24,25]. For example, the decay rate of fish eDNA in sediment is significantly lower than that in water (0.033%/h vs. 1.9%/h), and eDNA is 8–1800 times more concentrated in sediment than in water. This suggests that sediment eDNA can capture a broader range of species, including those that may be temporarily absent or less active in the water column.

### 4.2. Limitations of Traditional UVC and eDNA Methods

While the traditional UVC method is effective for detecting large and highly active organisms, they have limited capacity for detecting small or cryptic species [2,26]. In the present study, this limitation is partly due to the difficulty in capturing and identifying relatively small organisms such as polychaetes (belonging to the phylum Annelida), Chaetognatha, Gastrotricha, Nemertea, Platyhelminthes, and Arthropoda, which often require further taxonomic examination under a microscope. Additionally, environmental factors such as water turbidity in the subtidal zones can further reduce the effectiveness of the UVC method. However, traditional UVC remains valuable for certain taxa that may not be well-represented in eDNA samples, such as Bryozoa, which was undetectable by eDNA in our study but was identified through the UVC method.

The missed detection of Bryozoa highlights the need for further refinement of eDNA techniques. The COI primers used in this study failed to detect Bryozoa, indicating potential limitations in primer universality or suboptimal annealing temperature for PCR reactions. Additionally, the incomplete nature of current DNA sequence databases limits the accuracy of species-level identifications. Future research should focus on developing more universal primers and optimal PCR reactions, and expanding reference databases to improve the reliability and resolution of eDNA-based biodiversity assessments.

### 4.3. Complementary Nature of the Methods and Implications for Biodiversity Monitoring

In general, taxonomic compositions from the three methods shared relatively few taxa at the order level and below, and no common species was found among these methods (Figure 2c and Figure 3). The low overlap in species detected by the three methods underscores their complementary nature. Sediment eDNA provided the most comprehensive species inventory, detecting a significantly higher number of species compared to water eDNA and traditional UVC methods. However, the unique detection of certain taxa by the UVC method highlights the importance of integrating both eDNA and UVC approaches for a more complete assessment of biodiversity. This integration can help overcome the limitations of each method, such as primer bias in eDNA techniques and the challenges of species identification in traditional methods. Future biodiversity monitoring efforts should consider the complementary nature of these methods and the potential for further technological advancements to enhance the accuracy and efficiency of biodiversity assessments. This integrated approach will be crucial for informing effective conservation and management strategies in complex aquatic environments.

## 5. Conclusions

The present study provides a comprehensive evaluation of environmental DNA (eDNA) metabarcoding of COI and traditional underwater visual census (UVC) methods for assessing benthic macrofaunal diversity in subtidal zones. While taxonomic compositions from the three methods show similar patterns at the phylum level, they share relatively few taxa at the order level and below. Sediment eDNA emerged as a highly effective tool, particularly for detecting key benthic phyla such as Annelida and Arthropoda, due to its enhanced preservation and detection capabilities. However, traditional UVC remains crucial for identifying certain taxa, such as Bryozoa, which were undetectable by eDNA methods. The low overlap in species detected by these methods underscores their complementary nature, highlighting the necessity of integrating multiple approaches to achieve a more comprehensive and accurate biodiversity assessment. Future research should focus on refining eDNA techniques, such as developing more universal primers and expanding reference databases, to further enhance their applicability in biodiversity monitoring.

## Figures and Tables

**Table 1:** Species number of phyla using sediment and water eDNA and UVC methods.

Phylum	Sediment	Water	UVC
Annelida	22	8	1
Arthropoda	48	14	1
Bryozoa	0	0	5
Chaetognatha	1	4	0
Chordata	5	1	1
Cnidaria	26	32	7
Ctenophora	2	0	1
Echinodermata	5	4	4
Gastrotricha	6	1	0
Mollusca	37	19	17
Nematoda	3	0	0
Nemertea	2	2	0
Phoronida	1	0	0
Platyhelminthes	1	2	0
Porifera	7	7	2
total	166	94	39

## Data Availability

The raw sequence reads detected by eDNA are deposited in the NCBI Sequence Read Archive database with the BioProjects PRJNA1248872 and PRJNA1248901.

## Appendix A

**Table A1:** Species composition based on the sediment and water eDNA and UVC methods.

Species ID	Sediment	Water	UVC	Phylum	Class	Order	Family	Genus	Species
OTU_1958	1	1	0	Annelida	Polychaeta				Polychaeta sp. NB-Po509
OTU_1601	1	0	0	Annelida	Clitellata	Enchytraeida	Enchytraeidae	*Enchytronia*	*Enchytronia christenseni*
OTU_4793	1	0	0	Annelida	Polychaeta	Terebellida	Flabelligeridae	*Treadwellius*	*Treadwellius bifidus*
OTU_16546	1	0	0	Annelida	Polychaeta		Maldanidae	*Asychis*	*Asychis chilensis*
OTU_17049	1	0	0	Annelida	Clitellata	Crassiclitellata	Megascolecidae	*Perionyx*	*Perionyx rufulus*
OTU_5756	1	0	0	Annelida	Polychaeta	Phyllodocida	Nereididae	*Neanthes*	*Neanthes* sp. RP2011-N
OTU_7431	1	0	0	Annelida	Polychaeta	Phyllodocida	Nereididae	*Neanthes*	*Neanthes* sp. 1
OTU_4848	1	0	0	Annelida	Polychaeta		Orbiniidae	*Scoloplos*	*Scoloplos acmeceps*
OTU_2403	1	0	0	Annelida		Phascolosomatiformes	Phascolosomatidae	*Phascolosoma*	*Phascolosoma esculenta*
OTU_13606	1	0	0	Annelida	Polychaeta	Sabellida	Serpulidae	*Serpula*	*Serpula vermicularis*
OTU_14440	1	0	0	Annelida	Polychaeta	Sabellida	Serpulidae	*Pileolaria*	*Pileolaria* sp. 11BIOAK-0893
OTU_3016	1	0	0	Annelida	Polychaeta	Sabellida	Serpulidae	*Crucigera*	*Crucigera* sp. CMC01
OTU_331	1	0	0	Annelida	Polychaeta	Sabellida	Serpulidae	*Serpula*	*Serpula columbiana*
OTU_7545	1	0	0	Annelida	Clitellata	Lumbriculida	Sparganophilidae	*Sparganophilus*	*Sparganophilus* sp. MAC_SRS_S115
OTU_14076	1	0	0	Annelida	Polychaeta	Spionida	Spionidae	*Polydora*	*Polydora cornuta*
OTU_2533	1	0	0	Annelida	Polychaeta	Spionida	Spionidae	*Paraprionospio*	*Paraprionospio patiens*
OTU_16980	1	0	0	Annelida	Polychaeta	Terebellida	Terebellidae	*Thelepus*	*Thelepus* sp. CMC01
OTU_2402	1	0	0	Annelida	Polychaeta	Terebellida	Terebellidae	*Lysilla*	*Lysilla pacifica*
OTU_15614	1	0	0	Annelida	Polychaeta	Terebellida	Trichobranchidae	*Terebellides*	*Terebellides stroemii*
OTU_17438	1	0	0	Annelida	Polychaeta	Xenopneusta	Urechidae	*Urechis*	*Urechis unicinctus*
OTU_13481	1	0	0	Annelida	Polychaeta				Polychaeta sp. EBS61o-Po36
OTU_3837	1	0	0	Annelida	Polychaeta				Polychaeta sp. OPC-2015
OTU_12617	0	1	0	Annelida	Polychaeta	Sabellida	Serpulidae	*Protolaeospira*	*Protolaeospira eximia*
OTU_1436	0	1	0	Annelida	Clitellata	Rhynchobdellida	Glossiphoniidae	*Torix*	*Torix tukubana*
OTU_1486	0	1	0	Annelida	Clitellata	Crassiclitellata	Megascolecidae	*Amynthas*	*Amynthas exiguus*
OTU_2537	0	1	0	Annelida	Polychaeta	Terebellida	Terebellidae	*Loimia*	*Loimia arborea*
OTU_3294	0	1	0	Annelida	Polychaeta	Sabellida	Serpulidae	*Serpula*	*Serpula vermicularis*
OTU_518	0	1	0	Annelida	Polychaeta	Spionida	Trochochaetidae	*Trochochaeta*	*Trochochaeta multisetosa*
OTU_6200	0	1	0	Annelida	Polychaeta	Phyllodocida	Nereididae	*Nereis*	*Nereis* sp. CMC01
UVC_01	0	0	1	Annelida	Polychaeta	sabellida	Sabellidae		Sabellidae sp.
UVC_02	0	0	1	Arthropoda	Malacostraca	Decapoda	Paguridae	*Pagurus*	*Pagurus hedleyi*
OTU_2085	1	1	0	Arthropoda	Collembola	Entomobryomorpha	Entomobryidae	*Entomobrya*	*Entomobrya unostrigata*
OTU_13808	1	0	0	Arthropoda	Hexanauplia	Harpacticoida	Aegisthidae		Aegisthidae sp. DZMB479
OTU_8881	1	0	0	Arthropoda	Hexanauplia	Harpacticoida	Aegisthidae		Aegisthidae sp. 1
OTU_14428	1	0	0	Arthropoda	Malacostraca	Decapoda	Aeglidae	*Aegla*	*Aegla longirostri* complex sp. UFSM SG15
OTU_7458	1	0	0	Arthropoda	Hexanauplia	Sessilia	Archaeobalanidae	*Conopea*	*Conopea basicuneata*
OTU_11323	1	0	0	Arthropoda	Ichthyostraca	Arguloida	Argulidae	*Argulus*	*Argulus japonicus*
OTU_13718	1	0	0	Arthropoda	Malacostraca	Isopoda	Armadillidae	*Spherillo*	*Spherillo* sp. Kanzaki
OTU_5774	1	0	0	Arthropoda	Branchiopoda	Anostraca	Branchipodidae	*Parartemia*	*Parartemia serventyi*
OTU_8686	1	0	0	Arthropoda	Branchiopoda	Anostraca	Branchipodidae	*Parartemia*	*Parartemia* sp. 1
OTU_6592	1	0	0	Arthropoda	Malacostraca	Decapoda	Cambaridae	*Faxonius*	*Faxonius validus*
OTU_14411	1	0	0	Arthropoda	Malacostraca	Amphipoda	Chiltoniidae		Chiltoniidae sp. CU1
OTU_5711	1	0	0	Arthropoda	Hexanauplia	Sessilia	Chthamalidae	*Chthamalus*	*Chthamalus panamensis*
OTU_13431	1	0	0	Arthropoda	Ostracoda	Podocopida	Cyprididae	*Strandesia*	*Strandesia velhoi*
OTU_14178	1	0	0	Arthropoda	Ostracoda	Podocopida	Cyprididae	*Cyprideis*	*Cyprideis torosa*
OTU_9379	1	0	0	Arthropoda	Ostracoda	Podocopida	Darwinulidae	*Darwinula*	*Darwinula stevensoni*
OTU_14387	1	0	0	Arthropoda	Hexanauplia	Calanoida	Diaptomidae	*Hesperodiaptomus*	*Hesperodiaptomus arcticus*
OTU_4874	1	0	0	Arthropoda	Collembola	Symphypleona	Dicyrtomidae		Dicyrtomidae sp. BIOUG21696-E03
OTU_14442	1	0	0	Arthropoda	Collembola	Entomobryomorpha	Entomobryidae	*Entomobrya*	*Entomobrya decemfasciata*
OTU_5455	1	0	0	Arthropoda	Collembola	Entomobryomorpha	Entomobryidae	*Lepidocyrtus*	*Lepidocyrtus* sp. COLNO287-09
OTU_8097	1	0	0	Arthropoda	Collembola	Entomobryomorpha	Entomobryidae	*Entomobrya*	*Entomobrya* sp. HB0852_3
OTU_4780	1	0	0	Arthropoda	Malacostraca	Amphipoda	Eusiridae		Eusiridae sp. 06 MK-2021
OTU_4668	1	0	0	Arthropoda	Malacostraca	Decapoda	Grapsidae	*Grapsus*	*Grapsus albolineatus*
OTU_2814	1	0	0	Arthropoda	Malacostraca	Isopoda	Haploniscidae	*Mastigoniscus*	*Mastigoniscus* sp. NB-Iso41
OTU_14314	1	0	0	Arthropoda	Collembola	Poduromorpha	Hypogastruridae		Hypogastruridae sp. BIOUG23106-B05
OTU_7317	1	0	0	Arthropoda	Malacostraca	Isopoda	Ischnomesidae	*Ischnomesus*	*Ischnomesus* sp. lineage 7d JB-2020
OTU_7631	1	0	0	Arthropoda	Ostracoda	Podocopida	Loxoconchidae	*Microloxoconcha*	*Microloxoconcha ikeyai*
OTU_13364	1	0	0	Arthropoda	Malacostraca	Amphipoda	Lysianassidae	*Eurythenes*	*Eurythenes gryllus*
OTU_4425	1	0	0	Arthropoda	Malacostraca	Amphipoda	Lysianassidae	*Eurythenes*	*Eurythenes* sp. 1
OTU_4697	1	0	0	Arthropoda	Malacostraca	Amphipoda	Lysianassidae	*Eurythenes*	*Eurythenes* sp. 2A2 HR-2015
OTU_17634	1	0	0	Arthropoda	Hexanauplia	Harpacticoida	Miraciidae	*Psammotopa*	*Psammotopa* sp. n. SR-2019
OTU_6939	1	0	0	Arthropoda	Malacostraca	Mysida	Mysidae	*Paramesopodopsis*	*Paramesopodopsis rufa*
OTU_1240	1	0	0	Arthropoda	Collembola	Poduromorpha	Neanuridae	*Morulina*	*Morulina mackenziana*
OTU_2412	1	0	0	Arthropoda	Malacostraca	Decapoda	Oregoniidae	*Pleistacantha*	*Pleistacantha kannu*
OTU_1556	1	0	0	Arthropoda	Malacostraca	Decapoda	Palaemonidae	*Macrobrachium*	*Macrobrachium naiyanetri*
OTU_5707	1	0	0	Arthropoda	Malacostraca	Decapoda	Pandalidae	*Plesionika*	*Plesionika semilaevis*
OTU_9864	1	0	0	Arthropoda	Malacostraca	Decapoda	Pandalidae	*Pandalus*	*Pandalus montagui*
OTU_13528	1	0	0	Arthropoda	Diplopoda	Polyxenida	Polyxenidae		Polyxenidae sp. Biologic-POLX001
OTU_12157	1	0	0	Arthropoda	Malacostraca	Decapoda	Porcellanidae	*Petrolisthes*	*Petrolisthes rufescens*
OTU_13725	1	0	0	Arthropoda	Malacostraca	Decapoda	Portunidae	*Portunus*	*Portunus ventralis*
OTU_1312	1	0	0	Arthropoda	Collembola	Entomobryomorpha	Tomoceridae	*Tomocerus*	*Tomocerus ocreatus*
OTU_1352	1	0	0	Arthropoda	Malacostraca	Isopoda	Tylidae	*Tylos*	*Tylos ponticus*
OTU_17543	1	0	0	Arthropoda	Malacostraca	Amphipoda	Typhlogammaridae	*Typhlogammarus*	*Typhlogammarus* sp. ZH-2014
OTU_14472	1	0	0	Arthropoda	Hexanauplia	Sessilia	Verrucidae	*Metaverruca*	*Metaverruca recta*
OTU_13506	1	0	0	Arthropoda	Malacostraca	Amphipoda			Amphipoda sp. LPdivOTU157
OTU_17242	1	0	0	Arthropoda	Hexanauplia	Copepoda			Copepoda environmental sample
OTU_2669	1	0	0	Arthropoda	Hexanauplia	Copepoda			Copepoda sp. RR-2014
OTU_6511	1	0	0	Arthropoda					Arthropoda environmental sample
OTU_7414	1	0	0	Arthropoda	Hexanauplia	Harpacticoida			Harpacticoida sp. 10BIOBC-00681
OTU_11071	0	1	0	Arthropoda	Hexanauplia	Calanoida	Diaptomidae	*Allodiaptomus*	*Allodiaptomus mirabilipes*
OTU_11156	0	1	0	Arthropoda	Ostracoda	Podocopida	Cyprididae	*Cypretta*	*Cypretta campechensis*
OTU_15398	0	1	0	Arthropoda					Arthropoda sp. NZAC 03011539
OTU_15775	0	1	0	Arthropoda	Ostracoda	Podocopida	Candonidae	*Pseudocandona*	*Pseudocandona hartwigi*
OTU_17132	0	1	0	Arthropoda	Malacostraca	Euphausiacea	Euphausiidae	*Pseudeuphausia*	*Pseudeuphausia sinica*
OTU_1993	0	1	0	Arthropoda	Collembola	Symphypleona	Bourletiellidae	*Heterosminthurus*	*Heterosminthurus* sp. 2 JRD-2015
OTU_4032	0	1	0	Arthropoda	Malacostraca	Amphipoda	Stenothoidae	*Metopa*	*Metopa boeckii*
OTU_4984	0	1	0	Arthropoda	Hexanauplia	Siphonostomatoida			Siphonostomatoida sp. DZMB115
OTU_6169	0	1	0	Arthropoda	Branchiopoda	Diplostraca	Limnadiidae	*Limnadopsis*	*Limnadopsis* cf. *parvispinus*
OTU_6178	0	1	0	Arthropoda	Ostracoda	Podocopida	Cyprididae	*Ngarawa*	*Ngarawa* sp. 950.5
OTU_6301	0	1	0	Arthropoda	Malacostraca	Isopoda	Desmosomatidae		Desmosomatidae sp. NB-Iso234
OTU_8282	0	1	0	Arthropoda	Malacostraca	Isopoda	Ligiidae	*Ligia*	*Ligia baudiniana*
OTU_8283	0	1	0	Arthropoda	Malacostraca	Amphipoda	Niphargidae	*Niphargus*	*Niphargus parenzani*
UVC_03	0	0	1	Bryozoa	Gymnolaemata	Cheilostomatida	Membraniporidae	*Biflustra*	*Biflustra grandicella*
UVC_04	0	0	1	Bryozoa					Bryozoa sp.
UVC_05	0	0	1	Bryozoa	Gymnolaemata	Cheilostomatida	Bitectiporidae	*entapora*	*entapora fascialis*
UVC_31	0	0	1	Bryozoa	Stenolaemata	Cyclostomatida	Crisiidae	*Crisia*	*Crisia elongata*
UVC_33	0	0	1	Bryozoa	Cheilostomatida		Candidae	*Caberea*	*Caberea lata*
OTU_10240	1	1	0	Chaetognatha	Sagittoidea	Aphragmophora	Sagittidae	*Sagitta*	*Sagitta* sp. ZP304
OTU_36	0	1	0	Chaetognatha	Sagittoidea	Aphragmophora	Sagittidae	*Zonosagitta*	*Zonosagitta nagae*
OTU_93	0	1	0	Chaetognatha					Chaetognatha environmental sample
OTU_971	0	1	0	Chaetognatha					Chaetognatha environmental sample
OTU_14459	1	0	0	Chordata	Ascidiacea	Aplousobranchia	Didemnidae	*Didemnum*	*Didemnum candidum*
OTU_13738	1	0	0	Chordata	Thaliacea	Pyrosomata	Pyrosomatidae	*Pyrostremma*	*Pyrostremma spinosum*
OTU_13999	1	0	0	Chordata	Thaliacea	Salpida	Salpidae	*Cyclosalpa*	*Cyclosalpa* sp. USNM IZ 1449879
OTU_13033	1	0	0	Chordata	Ascidiacea	Stolidobranchia	Styelidae	*Cnemidocarpa*	*Cnemidocarpa verrucosa*
OTU_2807	1	0	0	Chordata	Ascidiacea	Stolidobranchia	Styelidae	*Cnemidocarpa*	*Cnemidocarpa verrucosa*
OTU_121	0	1	0	Chordata	Ascidiacea	Stolidobranchia	Styelidae	*Cnemidocarpa*	*Cnemidocarpa* sp. 1
UVC_06	0	0	1	Chordata	Ascidiacea				Ascidiacea sp.
UVC_30	0	0	0	Chordata	Ascidiacea	Stolidobranchia	Styelidae	*styela*	*styela canopus*
OTU_8074	1	1	0	Cnidaria	Hydrozoa	Leptothecata	Aequoreidae	*Aequorea*	*Aequorea* sp. USNM IZ 1450497
OTU_17794	1	1	0	Cnidaria	Hydrozoa	Anthoathecata	Candelabridae	*Candelabrum*	*Candelabrum cocksii*
OTU_1173	1	1	0	Cnidaria	Hydrozoa	Leptothecata	Clytiidae	*Clytia*	*Clytia paulensis*
OTU_1784	1	1	0	Cnidaria	Hydrozoa	Leptothecata	Clytiidae	*Clytia*	*Clytia gracilis*
OTU_16831	1	1	0	Cnidaria	Hydrozoa	Anthoathecata	Corynidae	*Coryne*	*Coryne* sp. JRH-2014
OTU_17154	1	0	0	Cnidaria	Anthozoa	Scleractinia	Acroporidae	*Acropora*	*Acropora* sp. IP0357
OTU_17856	1	0	0	Cnidaria	Hydrozoa	Anthoathecata	Bougainvilliidae	*Bougainvillia*	*Bougainvillia muscus*
OTU_2568	1	0	0	Cnidaria	Anthozoa	Scleractinia	Caryophylliidae	*Dasmosmilia*	*Dasmosmilia* cf. *lymani* MVK-2010
OTU_2663	1	0	0	Cnidaria	Anthozoa	Scleractinia	Caryophylliidae	*Rhizosmilia*	*Rhizosmilia robusta*
OTU_9057	1	0	0	Cnidaria	Scyphozoa	Rhizostomeae	Cassiopeidae	*Cassiopea*	*Cassiopea* sp. AUQLCBC
OTU_3574	1	0	0	Cnidaria	Hydrozoa	Anthoathecata	Corynidae	*Stauridiosarsia*	*Stauridiosarsia cliffordi*
OTU_13582	1	0	0	Cnidaria	Staurozoa	Stauromedusae	Craterolophidae	*Craterolophus*	*Craterolophus convolvulus*
OTU_3839	1	0	0	Cnidaria	Hydrozoa	Anthoathecata	Eudendriidae	*Eudendrium*	*Eudendrium carneum*
OTU_5607	1	0	0	Cnidaria	Hydrozoa	Leptothecata	Lafoeidae	*Lafoea*	*Lafoea dumosa*
OTU_10300	1	0	0	Cnidaria	Hydrozoa	Leptothecata	Laodiceidae	*Laodicea*	*Laodicea undulata*
OTU_13976	1	0	0	Cnidaria	Staurozoa	Stauromedusae	Lucernariidae	*Lucernaria*	*Lucernaria quadricornis*
OTU_17088	1	0	0	Cnidaria	Staurozoa	Stauromedusae	Lucernariidae		Lucernariidae sp. CCSMA208-10
OTU_10965	1	0	0	Cnidaria	Hydrozoa	Leptothecata	Obeliidae	*Obelia*	*Obelia dichotoma*
OTU_13515	1	0	0	Cnidaria	Hydrozoa	Leptothecata	Obeliidae	*Obelia*	*Obelia* sp. DNZ116
OTU_3127	1	0	0	Cnidaria	Hydrozoa	Anthoathecata	Oceaniidae	*Turritopsis*	*Turritopsis lata*
OTU_14409	1	0	0	Cnidaria	Hydrozoa	Anthoathecata	Pandeidae	*Leuckartiara*	*Leuckartiara* sp. PS-2018
OTU_13700	1	0	0	Cnidaria	Hydrozoa	Siphonophorae	Rhodaliidae	*Dendrogramma*	*Dendrogramma enigmatica*
OTU_4766	1	0	0	Cnidaria	Hydrozoa	Leptothecata	Sertulariidae	*Fraseroscyphus*	*Fraseroscyphus hozawai*
OTU_3775	1	0	0	Cnidaria	Anthozoa	Scleractinia	Siderastreidae	*Psammocora*	*Psammocora profundacella*
OTU_11341	1	0	0	Cnidaria	Scyphozoa	Semaeostomeae	Ulmaridae	*Aurelia*	*Aurelia* sp. IDPAJGB
OTU_8311	1	0	0	Cnidaria	Hydrozoa	Anthoathecata	Zancleidae	*Zanclea*	*Zanclea sango*
OTU_10172	0	1	0	Cnidaria	Hydrozoa	Leptothecata	Eirenidae	*Helgicirrha*	*Helgicirrha malayensis*
OTU_104	0	1	0	Cnidaria	Hydrozoa	Leptothecata	Clytiidae	*Clytia*	*Clytia hemisphaerica*
OTU_10944	0	1	0	Cnidaria	Hydrozoa	Anthoathecata	Corymorphidae	*Euphysa*	*Euphysa aurata*
OTU_11207	0	1	0	Cnidaria	Anthozoa	Actiniaria	Phymanthidae	*Phymanthus*	*Phymanthus* sp. IP0414
OTU_117	0	1	0	Cnidaria	Anthozoa	Alcyonacea	Plexauridae	*Euplexaura*	*Euplexaura* sp. A CSM-2013
OTU_15386	0	1	0	Cnidaria	Staurozoa	Stauromedusae	Haliclystidae	*Haliclystus*	*Haliclystus inabai*
OTU_15496	0	1	0	Cnidaria	Hydrozoa	Anthoathecata	Corynidae		Corynidae sp. BCnid2010-002
OTU_15730	0	1	0	Cnidaria	Hydrozoa	Anthoathecata	Asyncorinidae	*Asyncoryne*	*Asyncoryne ryniensis*
OTU_15819	0	1	0	Cnidaria	Hydrozoa	Siphonophorae	Prayidae	*Praya*	*Praya reticulata*
OTU_15894	0	1	0	Cnidaria	Scyphozoa	Rhizostomeae	Rhizostomatidae	*Rhopilema*	*Rhopilema hispidum*
OTU_15897	0	1	0	Cnidaria	Hydrozoa	Anthoathecata	Pandeidae	*Amphinema*	*Amphinema* sp. JRH-2012
OTU_16864	0	1	0	Cnidaria	Hydrozoa	Leptothecata	Plumulariidae	*Plumularia*	*Plumularia virginiae*
OTU_16960	0	1	0	Cnidaria	Hydrozoa	Anthoathecata	Bougainvilliidae	*Bougainvillia*	*Bougainvillia muscus*
OTU_17210	0	1	0	Cnidaria	Hydrozoa	Siphonophorae	Agalmatidae	*Marrus*	*Marrus* sp. BO-2021
OTU_17266	0	1	0	Cnidaria	Hydrozoa	Anthoathecata	Corynidae	*Coryne*	*Coryne* sp. JRH-2014
OTU_1954	0	1	0	Cnidaria	Hydrozoa	Leptothecata	Aglaopheniidae	*Aglaophenia*	*Aglaophenia tubulifera*
OTU_1962	0	1	0	Cnidaria	Hydrozoa	Leptothecata	Obeliidae	*Obelia*	*Obelia* sp. BFHL 2016
OTU_2471	0	1	0	Cnidaria	Hydrozoa	Anthoathecata	Pandeidae	*Amphinema*	*Amphinema* sp. PS-2017
OTU_2502	0	1	0	Cnidaria	Hydrozoa	Anthoathecata	Zancleidae	*Zanclea*	*Zanclea giancarloi*
OTU_3269	0	1	0	Cnidaria	Scyphozoa	Semaeostomeae	Cyaneidae	*Cyanea*	*Cyanea* sp. RUNNPEC
OTU_473	0	1	0	Cnidaria	Hydrozoa	Anthoathecata	Corynidae	*Coryne*	*Coryne* sp. JRH-2014
OTU_5038	0	1	0	Cnidaria	Hydrozoa	Leptothecata	Obeliidae	*Obelia*	*Obelia bidentata*
OTU_8072	0	1	0	Cnidaria	Anthozoa	Alcyonacea	Victorgorgiidae	*Victorgorgia*	*Victorgorgia* sp. 2 KMM-2015
OTU_8128	0	1	0	Cnidaria	Anthozoa	Scleractinia	Acroporidae	*Astreopora*	*Astreopora myriophthalma*
OTU_8132	0	1	0	Cnidaria	Scyphozoa	Semaeostomeae	Pelagiidae	*Chrysaora*	*Chrysaora lactea*
OTU_8177	0	1	0	Cnidaria	Scyphozoa	Coronatae	Nausithoidae	*Nausithoe*	*Nausithoe* sp. NHM_353
OTU_969	0	1	0	Cnidaria	Hydrozoa	Leptothecata	Obeliidae	*Obelia*	*Obelia* sp. MZUSP 3356
UVC_36	0	0	1	Cnidaria	Hexacorallia	Actiniaria	Actiniidae	*Paracondylactis*	*Paracondylactis sinensis*
UVC_07	0	0	1	Cnidaria	Octocorallia	Malacalcyonacea	Melithaeidae	*Melithaea*	*Melithaea formosa*
UVC_08	0	0	1	Cnidaria	Hexacorallia	Ceriantharia	Cerianthidae		Cerianthidae sp.
UVC_09	0	0	1	Cnidaria	Octocorallia	malacalcyonacea	Isididae	*Hicksonella*	*Hicksonella guishanensis*
UVC_10	0	0	1	Cnidaria	Octocorallia				Octocorallia sp.
UVC_11	0	0	1	Cnidaria	Hexacorallia	Actiniaria	Actiniidae	*Anthopleura*	*Anthopleura japonica*
UVC_12	0	0	1	Cnidaria	Hexacorallia	Actiniaria	Actiniidae	*Anthopleura*	*Anthopleura inornata*
OTU_13644	1	0	1	Ctenophora	Nuda	Beroida	Beroidae	*Beroe*	*Beroe ovata*
OTU_14325	1	0	0	Ctenophora	Nuda	Beroida	Beroidae	*Beroe*	*Beroe* sp. 2
UVC_34	0	0	1	Echinodermata	Holothuroidea	Dendrochirotida	Cucumariidae	*Actinia*	*Actinia equina*
OTU_8133	0	0	1	Echinodermata	Asteroidea	Forcipulatida	Asteriidae	*Stichaster*	*Stichaster striatus*
UVC_13	0	0	1	Echinodermata	Echinoidea	Camarodonta	Echinometridae	*Anthocidaris*	*Anthocidaris crassispina*
UVC_14	0	0	1	Echinodermata	Echinoidea	Camarodonta	Temnopleuridae	*Temnopleurus*	*Temnopleurus hardwickii*
OTU_4816	1	0	0	Echinodermata	Ophiuroidea	Euryalida	Gorgonocephalidae	*Astrodendrum*	*Astrodendrum* sp. NSMT:E-6273
OTU_324	1	0	0	Echinodermata	Ophiuroidea	Amphilepidida	Ophiactidae	*Ophiactis*	*Ophiactis perplexa*
OTU_2974	1	0	0	Echinodermata	Ophiuroidea	Amphilepidida	Ophiolepididae	*Ophiolepis*	*Ophiolepis irregularis*
OTU_5418	1	0	0	Echinodermata	Ophiuroidea	Amphilepidida	Ophiotrichidae	*Ophiothrix*	*Ophiothrix spiculata*
OTU_14449	1	0	0	Echinodermata	Crinoidea	Cyrtocrinida	Sclerocrinidae	*Neogymnocrinus*	*Neogymnocrinus richeri*
OTU_11136	0	1	0	Echinodermata	Crinoidea	Comatulida	Antedonidae	*Antedon*	*Antedon loveni*
OTU_16102	0	1	0	Echinodermata	Ophiuroidea	Euryalida	Gorgonocephalidae	*Astrothrombus*	*Astrothrombus chrysanthi*
OTU_466	0	1	0	Echinodermata	Crinoidea	Comatulida	Antedonidae	*Antedon*	*Antedon loveni*
OTU_8133	0	1	0	Echinodermata	Asteroidea	Forcipulatida	Asteriidae	*Stichaster*	*Stichaster striatus*
OTU_17372	1	0	0	Gastrotricha		Chaetonotida	Chaetonotidae	*Chaetonotus*	*Chaetonotus* aff. *gelidus* MK-2019
OTU_3701	1	0	0	Gastrotricha		Chaetonotida	Chaetonotidae	*Aspidiophorus*	*Aspidiophorus* sp. n. MK-2019
OTU_4126	1	0	0	Gastrotricha		Chaetonotida	Chaetonotidae	*Aspidiophorus*	*Aspidiophorus* sp. 1
OTU_4761	1	0	0	Gastrotricha		Chaetonotida	Chaetonotidae	*Chaetonotus*	*Chaetonotus* sp. 2 MK-2019
OTU_6356	1	0	0	Gastrotricha		Chaetonotida	Chaetonotidae	*Chaetonotus*	*Chaetonotus* sp. 1
OTU_7757	1	0	0	Gastrotricha		Chaetonotida	Chaetonotidae	*Aspidiophorus*	*Aspidiophorus* sp. n. MK-2019
OTU_15575	0	1	0	Gastrotricha		Macrodasyida	Thaumastodermatidae	*Tetranchyroderma*	*Tetranchyroderma* sp. 3 TK-2011
OTU_1292	1	1	0	Mollusca	Gastropoda	Cycloneritida	Neritidae	*Neritina*	*Neritina* sp. Suji
OTU_1469	1	1	0	Mollusca	Gastropoda	Systellommatophora	Onchidiidae	*Melayonchis*	*Melayonchis tillieri*
OTU_3647	1	1	0	Mollusca	Gastropoda		Pachychilidae	*Sulcospira*	*Sulcospira paludiformis*
OTU_94	1	1	0	Mollusca	Gastropoda		Planorbidae	*Biomphalaria*	*Biomphalaria straminea*
OTU_6172	1	1	0	Mollusca	Gastropoda	Neogastropoda	Terebridae	*Myurella*	*Myurella affinis*
OTU_10351	1	0	0	Mollusca	Gastropoda	Architaenioglossa	Ampullariidae	*Pomacea*	*Pomacea catemacensis*
OTU_14521	1	0	0	Mollusca	Gastropoda	Architaenioglossa	Ampullariidae	*Pomacea*	*Pomacea canaliculata*
OTU_7625	1	0	0	Mollusca	Gastropoda	Aplysiida	Aplysiidae	*Aplysia*	*Aplysia fasciata*
OTU_13638	1	0	0	Mollusca	Gastropoda	Trochida	Calliostomatidae	*Calliostoma*	*Calliostoma iridium*
OTU_14338	1	0	0	Mollusca	Gastropoda	Stylommatophora	Camaenidae	*Xanthomelon*	*Xanthomelon interpositum*
OTU_14515	1	0	0	Mollusca	Gastropoda	Stylommatophora	Camaenidae	*Satsuma*	*Satsuma jacobii*
OTU_2134	1	0	0	Mollusca	Gastropoda	Stylommatophora	Camaenidae	*Satsuma*	*Satsuma hemihelvus*
OTU_5901	1	0	0	Mollusca	Gastropoda	Stylommatophora	Camaenidae	*Satsuma*	*Satsuma* sp. SPW-2007
OTU_9949	1	0	0	Mollusca	Gastropoda	Stylommatophora	Clausiliidae	*Montenegrina*	*Montenegrina helvola*
OTU_736	1	0	0	Mollusca	Gastropoda	Neogastropoda	Costellariidae	*Pusia*	*Pusia savignyi*
OTU_6464	1	0	0	Mollusca	Gastropoda	Nudibranchia	Cuthonellidae	*Cuthonella*	*Cuthonella punicea*
OTU_4617	1	0	0	Mollusca	Gastropoda	Architaenioglossa	Diplommatinidae	*Diplommatina*	*Diplommatina* sp. nov. AG MS-2010
OTU_6514	1	0	0	Mollusca	Gastropoda	Nudibranchia	Dotidae	*Doto*	*Doto coronata*
OTU_10095	1	0	0	Mollusca	Gastropoda	Ellobiida	Ellobiidae	*Ellobium*	*Ellobium scheepmakeri*
OTU_3788	1	0	0	Mollusca	Gastropoda	Lepetellida	Fissurellidae	*Fissurella*	*Fissurella* sp. SWA-2011
OTU_2686	1	0	0	Mollusca	Gastropoda	Stylommatophora	Geomitridae	*Xerocrassa*	*Xerocrassa ponsi*
OTU_4632	1	0	0	Mollusca	Gastropoda	Littorinimorpha	Hydrobiidae	*Marstonia*	*Marstonia lustrica*
OTU_14429	1	0	0	Mollusca	Gastropoda	Neogastropoda	Muricidae	*Concholepas*	*Concholepas concholepas*
OTU_9579	1	0	0	Mollusca	Gastropoda	Neogastropoda	Muricidae	*Drupa*	*Drupa morum*
OTU_14543	1	0	0	Mollusca	Gastropoda	Cycloneritida	Neritidae	*Nerita*	*Nerita atramentosa*
OTU_1864	1	0	0	Mollusca	Gastropoda	Cycloneritida	Neritidae	*Neritina*	*Neritina dilatata*
OTU_5511	1	0	0	Mollusca	Cephalopoda	Teuthida	Ommastrephidae	*Dosidicus*	*Dosidicus gigas*
OTU_10283	1	0	0	Mollusca	Gastropoda	Systellommatophora	Onchidiidae	*Paromoionchis*	*Paromoionchis daemelii*
OTU_13627	1	0	0	Mollusca	Gastropoda	Systellommatophora	Onchidiidae	*Onchidella*	*Onchidella marginata*
OTU_14456	1	0	0	Mollusca	Gastropoda		Pachychilidae	*Sulcospira*	*Sulcospira paludiformis*
OTU_1546	1	0	0	Mollusca	Bivalvia	Unionida	Unionidae	*Middendorffinaia*	*Middendorffinaia mongolica*
OTU_4294	1	0	0	Mollusca	Bivalvia	Unionida	Unionidae	*Pleuronaia*	*Pleuronaia barnesiana*
OTU_4892	1	0	0	Mollusca	Bivalvia	Unionida	Unionidae	*Toxolasma*	*Toxolasma lividus*
OTU_7039	1	0	0	Mollusca	Bivalvia	Unionida	Unionidae	*Aculamprotula*	*Aculamprotula fibrosa*
OTU_10224	1	0	0	Mollusca	Gastropoda	Littorinimorpha	Vermetidae	*Dendropoma*	*Dendropoma gregarium*
OTU_5629	1	0	0	Mollusca	Gastropoda	Littorinimorpha	Vermetidae	*Dendropoma*	*Dendropoma platypus*
OTU_14128	1	0	0	Mollusca	Bivalvia				Bivalvia environmental sample
OTU_12851	0	1	0	Mollusca	Gastropoda	Stylommatophora	Camaenidae	*Satsuma*	*Satsuma hemihelvus*
OTU_15803	0	1	0	Mollusca	Bivalvia	Galeommatida	Galeommatidae	*Galeomma*	*Galeomma turtoni*
OTU_1980	0	1	0	Mollusca	Gastropoda	Stylommatophora	Geomitridae	*Candidula*	*Candidula* sp. AR_BC7338
OTU_3248	0	1	0	Mollusca	Gastropoda	Neogastropoda	Terebridae	*Hastula*	*Hastula matheroniana*
OTU_3959	0	1	0	Mollusca	Gastropoda		Physidae	*Physella*	*Physella acuta*
OTU_4408	0	1	0	Mollusca	Gastropoda	Stylommatophora	Helicidae	*Eobania*	*Eobania vermiculata*
OTU_470	0	1	0	Mollusca	Gastropoda		Plakobranchidae	*Thuridilla*	*Thuridilla vatae*
OTU_6184	0	1	0	Mollusca	Gastropoda	Cycloneritida	Phenacolepadidae	*Plesiothyreus*	*Plesiothyreus newtoni*
OTU_6194	0	1	0	Mollusca	Bivalvia	Unionida	Unionidae	*Hyriopsis*	*Hyriopsis bialatus* complex sp. 2 PP-2017
OTU_6526	0	1	0	Mollusca	Gastropoda	Stylommatophora	Clausiliidae	*Montenegrina*	*Montenegrina umbilicata*
OTU_7189	0	1	0	Mollusca	Gastropoda	Stylommatophora	Camaenidae	*Rhagada*	*Rhagada angulata*
OTU_8077	0	1	0	Mollusca	Gastropoda	Siphonariida	Siphonariidae	*Siphonaria*	*Siphonaria lessonii*
OTU_86	0	1	0	Mollusca	Gastropoda	Littorinimorpha	Hydrobiidae	*Hauffenia*	*Hauffenia subpiscinalis*
OTU_965	0	1	0	Mollusca	Bivalvia	Galeommatida	Galeommatidae	*Galeomma*	*Galeomma turtoni*
UVC_37	0	0	1	Mollusca	Bivalvia	Mytilida	Mytilidae	*Perna*	*Perna viridis*
UVC_38	0	0	1	Mollusca	Bivalvia	Mytilida	Modiolidae	*Vignadula*	*Vignadula atrata*
UVC_15	0	0	1	Mollusca	Cephalopoda	Sepiida	Sepiidae	*Sepiella*	*Sepiella maindroni de Rochebrune*
UVC_16	0	0	1	Mollusca	Gastropoda	Trochidae	Calliostomatidae	*Tristichotrochus*	*Tristichotrochus unicum*
UVC_17	0	0	1	Mollusca	Gastropoda	Trochidae	Trochidae	*Monodonta*	*Monodonta labio*
UVC_18	0	0	1	Mollusca	Gastropoda	Littorinimorpha	Ovulidae		Ovulidae sp.
UVC_19	0	0	1	Mollusca	Gastropoda	Littorinimorpha	Ovulidae	*Cuspivolva*	*Cuspivolva formosa*
UVC_20	0	0	1	Mollusca	Gastropoda	Neogastropoda	Buccinidae	*Cantharus*	*Cantharus cecillei*
UVC_21	0	0	1	Mollusca	Gastropoda	Neogastropoda	Cymatiidae	*Gyrineum*	*Gyrineum natator*
UVC_22	0	0	1	Mollusca	Gastropoda	Neogastropoda	Muricidae	*Chicoreus*	*Chicoreus asianus*
UVC_23	0	0	1	Mollusca	Gastropoda	Neogastropoda	Muricidae	*Reishia*	*Reishia clavigera*
UVC_24	0	0	1	Mollusca	Gastropoda	Neogastropoda	Muricidae	*Reishia*	*Reishia luteostoma*
UVC_25	0	0	1	Mollusca	Gastropoda	Neogastropoda	Nassariidae	*Nassarius*	*Nassarius hepaticus*
UVC_26	0	0	1	Mollusca	Gastropoda	Nudibranchia			Nudibranchia sp.
UVC_27	0	0	1	Mollusca	Gastropoda	Nudibranchia	Facelinidae	*Sakuraeolis*	*Sakuraeolis sakuracea*
UVC_28	0	0	1	Mollusca	Gastropoda	Nudibranchia	Dendrodorididae	*Doriopsilla*	*Doriopsilla* sp.
UVC_32	0	0	1	Mollusca	Gastropoda		Pyramidellidae	*Odostomia*	*Odostomia subangulata*
OTU_7430	1	0	0	Nematoda	Chromadorea	Strongylida	Ancylostomatidae	*Ancylostoma*	*Ancylostoma ceylanicum*
OTU_7275	1	0	0	Nematoda	Chromadorea	Desmodorida	Desmodoridae	*Metachromadora*	*Metachromadora* sp. 2AMA
OTU_13801	1	0	0	Nematoda	Chromadorea	Rhabditida	Rhabditidae	*Caenorhabditis*	*Caenorhabditis inopinata*
OTU_8772	1	0	0	Nemertea	Pilidiophora	Heteronemertea	Baseodiscidae	*Baseodiscus*	*Baseodiscus* sp. 2 SA-2011
OTU_2470	1	0	0	Nemertea	Palaeonemertea		Tubulanidae	*Tubulanus*	*Tubulanus polymorphus*
OTU_17313	0	1	0	Nemertea	Enopla	Monostilifera			Monostilifera sp. MCZ IZ 45646
OTU_502	0	1	0	Nemertea	Enopla	Monostilifera	Amphiporidae	*Amphiporus*	*Amphiporus cruentatus*
OTU_17155	1	0	0	Phoronida					*Phoroniformea* sp. larval OTU P1
OTU_435	1	1	0	Platyhelminthes	Trematoda	Diplostomida	Diplostomidae	*Crassiphiala*	*Crassiphiala* sp. Lineage 2
OTU_16856	0	1	0	Platyhelminthes	Rhabditophora	Tricladida	Geoplanidae	*Microplana*	*Microplana* sp. MOTU37
OTU_897	1	0	0	Porifera	Demospongiae	Bubarida	Dictyonellidae	*Scopalina*	*Scopalina lophyropoda*
OTU_7359	1	0	0	Porifera	Demospongiae	Suberitida	Halichondriidae	*Ciocalypta*	*Ciocalypta* sp. DE-2012
OTU_8918	1	0	0	Porifera	Demospongiae	Haplosclerida	Niphatidae	*Amphimedon*	*Amphimedon queenslandica*
OTU_7393	1	0	0	Porifera	Homoscleromorpha	Homosclerophorida	Plakinidae	*Plakina*	*Plakina coerulea*
OTU_9873	1	0	0	Porifera	Homoscleromorpha	Homosclerophorida	Plakinidae	*Plakina*	*Plakina* sp. 1
OTU_7613	1	0	0	Porifera	Demospongiae	Axinellida	Raspailiidae		Raspailiidae sp. Po.25592
OTU_9606	1	0	0	Porifera	Demospongiae	Tetractinellida	Tetillidae	*Cinachyrella*	*Cinachyrella alloclada*
OTU_10893	0	1	0	Porifera	Demospongiae	Suberitida	Suberitidae	*Aaptos*	*Aaptos nuda*
OTU_15892	0	1	0	Porifera	Demospongiae	Suberitida	Halichondriidae		Halichondriidae sp. UCMPWC1015
OTU_16058	0	1	0	Porifera	Demospongiae	Poecilosclerida	Mycalidae	*Mycale*	*Mycale mirabilis*
OTU_16093	0	1	0	Porifera					Porifera sp. Sp08
OTU_17151	0	1	0	Porifera	Demospongiae	Tetractinellida	Tetillidae	*Cinachyrella*	*Cinachyrella australiensis*
OTU_2563	0	1	0	Porifera	Demospongiae	Tethyida	Hemiasterellidae	*Adreus*	*Adreus fascicularis*
OTU_5032	0	1	0	Porifera	Demospongiae	Haplosclerida	Chalinidae	*Haliclona*	*Haliclona* sp. P2MK
UVC_35	0	0	1	Porifera	Demospongiae	Tethyida	Tethyidae	*Tethya*	*Tethya aurantium*
UVC_29	0	0	1	Porifera	Demospongiae	Tethyida	Tethyidae	*Tethya*	*Tethya* sp. 1
